# The impact of anemia and blood transfusion on mortality after open abdominal surgery in the elderly

**DOI:** 10.1007/s00423-023-03122-w

**Published:** 2023-11-01

**Authors:** Henrik Buhl, Astrid Nørgaard, Astrid Otkjaer, Lars Nannestad Jørgensen, Henrik Løvendahl Jørgensen

**Affiliations:** 1 Department of Clinical Biochemistry, Hvidovre Hospital, University of Copenhagen, Kettegård Alle 30, 2650 Hvidovre, Denmark; 2 Pharmacosmos A/S, Rørvangsvej 30, 4300 Holbaek, Denmark; 3 Digestive Disease Center, Bispebjerg Hospital, University of Copenhagen, 2400 Copenhagen, NV Denmark; 4 Department of Clinical Medicine, University of Copenhagen, 2200 Copenhagen N, Denmark

**Keywords:** Anemia, Blood transfusion, Open abdominal surgery

## Abstract

**Background:**

Major abdominal surgery is associated with considerable mortality in the elderly. Anemia has been linked to increased mortality in other types of surgery, such as hip and cardiac surgery. This study aimed to assess the impact of preoperative anemia on mortality in the elderly undergoing major abdominal surgery, and how allogeneic red cell blood transfusion influences mortality in these patients.

**Materials and methods:**

We conducted a single-center, register-based retrospective study on patients, who were aged beyond 60 years and underwent one of 81 open abdominal surgical procedures. Patients operated on during the period from January 1, 2000, to May 31, 2013, were consecutively identified in the Danish National Patient Registry. Plasma hemoglobin was measured within 30 days prior to surgery and the primary endpoint was 30-day postoperative mortality. Information about patient transfusions from the hospital blood bank was available from 1998 to 2010.

**Results:**

A total of 3199 patients were included of whom 85% underwent emergency surgery. The total mortality after 30 days was 20%. The median preoperative hemoglobin value of survivors was 7.7 mmol/L vs 6.9 mmol/L in those who died. The difference in hemoglobin values, between those who survived or died, decreased from the pre- to the post-operative phase. The 30-day postoperative mortality was 28%, 20%, and 12% in patients with a preoperative hemoglobin level in the lower, median, and upper quartile respectively. Transfusion therapy was associated with higher postoperative mortality, except in patients with very low hemoglobin values.

**Conclusion:**

Preoperative anemia has a clear association with surgically related mortality. The distribution of hemoglobin values in patients with a fatal outcome differs significantly from that of survivors. Red cell transfusion is associated with increased mortality, except in patients with very low hemoglobin values which supports recent guidelines suggesting a restrictive transfusion strategy.

## Introduction

The incidence of open emergency abdominal surgery has been reported to 30.4 cases per 100,000 person-years with a 30-day postoperative mortality of 19% [1]. To lower mortality related to open abdominal surgery, it is necessary to understand both patient characteristics and treatments which contribute to the risk of dying. Multiple risk score models have been developed for surgery, but the impact of underlying conditions such as preoperative anemia on mortality has only been scarcely studied excluding anemia as a parameter from many risk models [2].

Our focus was to investigate the effects of preoperative hemoglobin levels on the outcomes of open abdominal surgery. Anemia is a frequent abnormality in the surgical population occurring in up to 29% of patients before surgery[3]. In a study on general non-cardiac surgery, anemia was associated with a 42% increased odds ratio of dying within the first 30 postoperative days [3]. The impact of preoperative hemoglobin levels on mortality after hip and heart surgery is well-studied [4]. A meta-analysis on hip surgery found anemia associated with a pooled mortality odds ratio of 2.78 [5].

The poor prognosis associated with preoperative anemia would make it logical to consider blood transfusion but three trials involving a total of 1965 patients presenting with acute upper gastrointestinal bleeding reported lower mortality following a restrictive transfusion threshold compared with a liberal transfusion threshold [6]. A randomized controlled study of 2016 patients at increased cardiovascular risk found no benefits of liberal postoperative transfusions [7]. Likewise, a study on blood transfusion in emergency abdominal surgery in Denmark found that transfusions were associated with poorer clinical outcomes [8]. Furthermore, several studies have documented that allogeneic transfusion is associated with circulatory overload, immunosuppression, postoperative infection, thromboembolic complications, increased length of hospital stay, recurrence of colorectal cancer, and consequently cost increase [9–11].

The aim of this study was to assess and discuss the impact of anemia and blood transfusions on mortality in open abdominal surgery. We also aimed to develop a model to compare the mortality risk in these patients across different preoperative hemoglobin levels in blood.

## Methods

### Patients

The Danish National Patient Registry collects data on all major surgical interventions performed in Denmark. From this registry, we extracted data on all patients who were aged more than 60 years and underwent open abdominal surgery at the Digestive Disease Center, Bispebjerg Hospital, Denmark, from January 1, 2000, to May 31, 2013, *n* = 4177. A total of 81 different types of surgery according to the NOMESCO Classification of Surgical Procedures[12] were included. Only the primary (most important) surgical procedure of an intervention and the first surgical intervention during the inclusion period were assessed. In Denmark, all citizens are registered with a unique civil registration number, enabling person-level linkage across nationwide registers. This enables virtually complete long-term follow-up as regards mortality and emigration. Patients with a temporary civil registration number, missing information regarding surgical priority, or lacking concomitant measurements of hemoglobin were excluded.

**Fig. 1:**
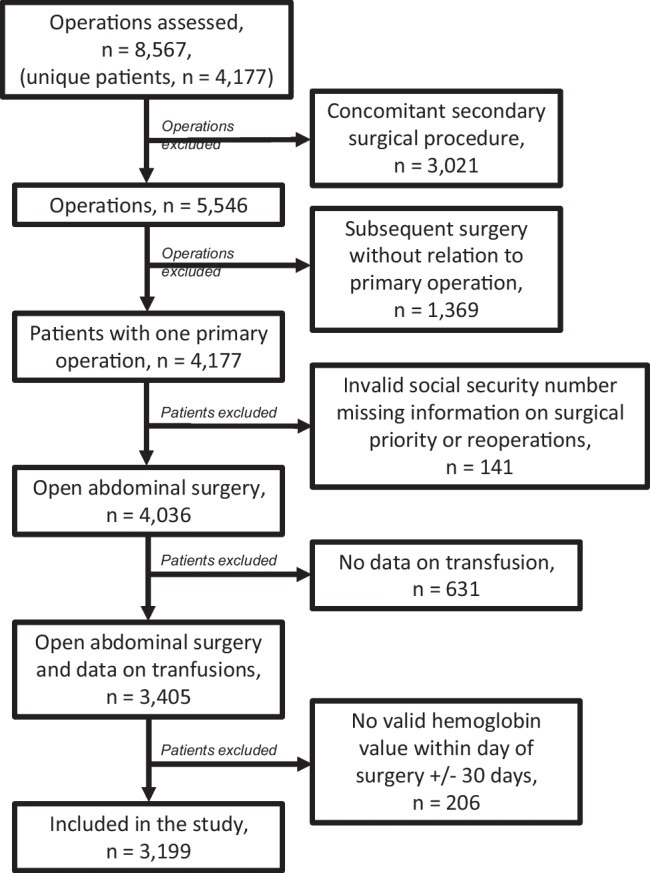
Study flow chart

The algorithm for patient selection is depicted in Fig. 1.

**Fig. 2:**
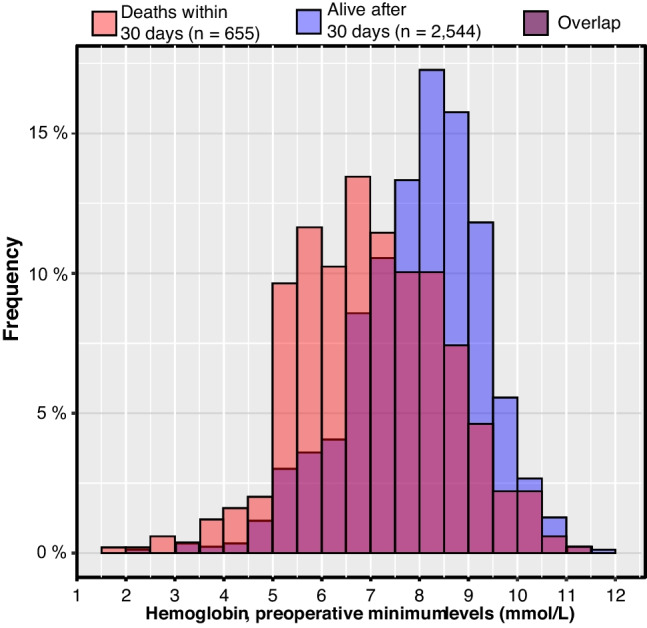
Distribution of patients versus minimum hemoglobin level preoperatively

In the top of the figure, the included surgical patients in the period of January 1, 2000, to May 31, 2013, can be seen. Below that, the number of patients who received transfusion is shown. We have data on transfusions from beginning of January 1998 to the end of December 2010. The final patients left for analysis were those from January 2000 until the end of December 2010 for whom we have complete data on surgical procedures, hemoglobin assessments, and transfusion therapy (Fig. 2).

### Hemoglobin level

Minimum and maximum hemoglobin values were obtained pre-, peri-, and post-operatively. Preoperative hemoglobin referred to the period within 30 days preceding the operation. Intraoperative was defined as sampling on the day of operation. The term postoperative covered the first 30 postoperative days. In cases with more than one measurement of hemoglobin either within 30 days prior or after the operation, the minimum and maximum values were recorded. Measurements were obtained from the local laboratory system. The normal range for plasma hemoglobin was 7.3–9.5 mmol/L for women and 8.3–10.5 mmol/L for men. The data obtained besides biochemical markers were time of hospital admission and discharge, priority of admission/surgery (elective vs emergency), type of operation, sex, and 30-day mortality.

### Transfusion data

Transfusion was defined as allogeneic transfusion of at least one unit of packed red cells. Red cell units were checked out of the hospital blood bank by scanning a bar code on the unit, which simultaneously recorded the date, the recipient identity, and the ISBT-128 identity code of the blood component type in the blood bank database. By national law, the handling of every issued blood component was recorded in the database, resulting in at least 99% traceability from donor to patient. The red blood cell transfusions were categorized as pre-, peri-, or post-operative according to the period of administration.

### Statistical analysis

The primary endpoint was 30-day mortality. Data analysis was performed using R statistical software [13]. The data was analyzed to create smoothed curves of mortality by fitting a locally estimated scatterplot smoothing curve (LOESS) to the data. The span parameter of the LOESS function was left at the default 0.75. Differences between baseline values in the cohort were analyzed using parametric or categorical statistical methods as appropriate. Continuous and categorical data were compared using unpaired *t*-test and chi-square test, respectively.

The prediction model was built using logistic regression, outputting the probability of death within 30 days. The parameters for the model were chosen using the stepwise procedure of *R*; using Akaike information criterion values to select parameters, all parameters had a *P*-value of < 0.05 with respect to predicting 30-day mortality postoperatively. After selection of parameters, receiver operating characteristics (ROC) were used to analyze the sensitivity and specificity of each component of the final model. The visualization was done using the ggplot2 package [14].

### Approvals

Permission for this study was granted by the Danish Data Protection Agency (ID: 2007–58-0015). No permission from the regional Ethical Committee was needed because the study was registry-based.

## Results

### The cohort

The basic characteristics of the 3199 included patients with a mean age of 76.6 years. The 30-day postoperative mortality was 655/3199 (20.5%). The characteristics of the groups differed between survivors and those who died. The mean preoperative hemoglobin was 7.5 mmol/L in survivors and 7.0 mmol/L in those who died (*P* < 0.001). The corresponding rates of emergency surgery were 61% and 92% (*P* < 0.001) (Table 1).

**Table 1:** Patient characteristics

	Alive after 30 days, *n* = 2544	Deaths within 30 days, *n* = 655	*P*	No transfusion, *n* = 1844	Transfusion, *n* = 1355	*P*
Age (SD), years	75.5 (9.1)	80.9 (8.2)	< 0.0001	74.9 (9.3)	78.8 (8.7)	< 0.0001
Females/males, *n* (%)	1564 (61.5)/980 (38.5)	413 (63.4)/240 (36.6)	0.4	1090 (59.1)/754 (40.9)	889 (65.6)/466 (34.4)	0.0002
Acute surgery/elective surgery, *n* (%)	1560 (61.3)/984 (38.7)	600 (91.6)/55 (8.4)	< 0.0001	1149 (62.3)/695 (37.7)	1011 (74.6)/344 (25.4)	< 0.0001
Hemoglobin mean level, mmol/L (SD)						
Minimum preoperative	7.5 (1.4)	7.0 (1.9)	< 0.0001	8.1 (1.2)	6.6 (1.4)	< 0.0001
Maximum preoperative	8.1 (1.2)	7.9 (1.3)	0.001	8.5 (1.1)	7.5 (1.2)	< 0.0001
Minimum perioperative	7.5 (1.4)	7.2 (1.5)	< 0.0001	8.1 (1.2)	6.7 (1.4)	< 0.0001
Maximum perioperative	7.7 (1.4)	7.5 (1.4)	0.02	8.2 (1.2)	7.0 (1.3)	< 0.0001
Minimum postoperative	6.4 (1.0)	6.4 (1.1)	0.7	6.7 (0.9)	6.0 (1.0)	< 0.0001
Maximum postoperative	7.6 (0.9)	7.6 (1.0)	0.4	7.6 (0.9)	7.6 (0.9)	0.05
Volume of red blood cell transfusion, units (SD)						
Total	1.1 (2.3)	2.1 (2.9)	< 0.0001	0	3.1	–
Preoperative	0.2 (1.0)	0.3 (1.3)	0.007	0	0.5	–
Perioperative	0.5 (1.2)	1.1 (1.8)	< 0.0001	0	1.5	–
Postoperative	0.4 (1.2)	0.6 (1.3)	0.002	0	1.1	–
Surgical category, *n* (%)						
Gastroduodenal	206 (8.1)	128 (19.5)		125 (6.8)	209 (15.4)	
Biliary	185 (7.3)	13 (2.0)		138 (7.5)	60 (4.4)	
Small bowel	175 (6.9)	66 (10.1)		144 (7.8)	97 (7.2)	
Appendix	152 (6.0)	17 (2.6)	< 0.0001	150 (8.1)	19 (1.4)	< 0.0001
Colon	968 (38.1)	225 (34.4)		603 (32.7)	590 (43.5)	
Rectum	400 (15.7)	46 (7.0)		240 (13.0)	206 (15.2)	
Miscellaneous*	458 (18.0)	160 (24.4)		444 (24.1)	174 2.8)	

*Herniotomy, adhesiolysis, splenectomy, or diagnostic procedures

### Mortality status depending on preoperative hemoglobin level

The median preoperative minimum hemoglobin value of survivors was 7.7 mmol/L vs 6.9 mmol/L in those who died. The corresponding median minimum hemoglobin levels in the intraoperative phase was 7.6 mmol/L and 7.2 mmol/L, respectively. In the postoperative phase, the median minimum hemoglobin value was 6.3 mmol/L in both groups.

**Fig. 3:**
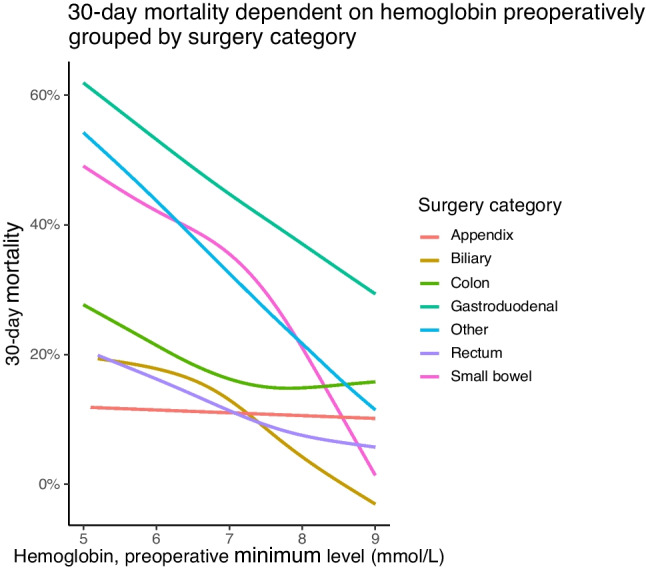
Mortality versus minimum preoperative hemoglobin level. A smoothed curve of mortality fitted versus the preoperative minimum hemoglobin level. Patients are grouped according to the surgery type they had. *Other includes herniotomy, adhesiolysis, splenectomy, and surgery for diagnostic purposes

The 30-day mortality correlated negatively with minimum preoperative hemoglobin levels (Fig. 3), the Pearson coefficient was − 0.15 (*P* < 0.001). Patients with a preoperative hemoglobin measurement in the lowest quarter progressing through to the highest quarter had a 30-day mortality of 28%, 19%, 15%, and 12% respectively.

### Transfusion rates

In total, 45% of women and 38% of men were transfused (*P* < 0.002). There was no significant difference in the amount of blood transfused between genders. Patients received an average of 1.3 portions of blood.

**Fig. 4:**
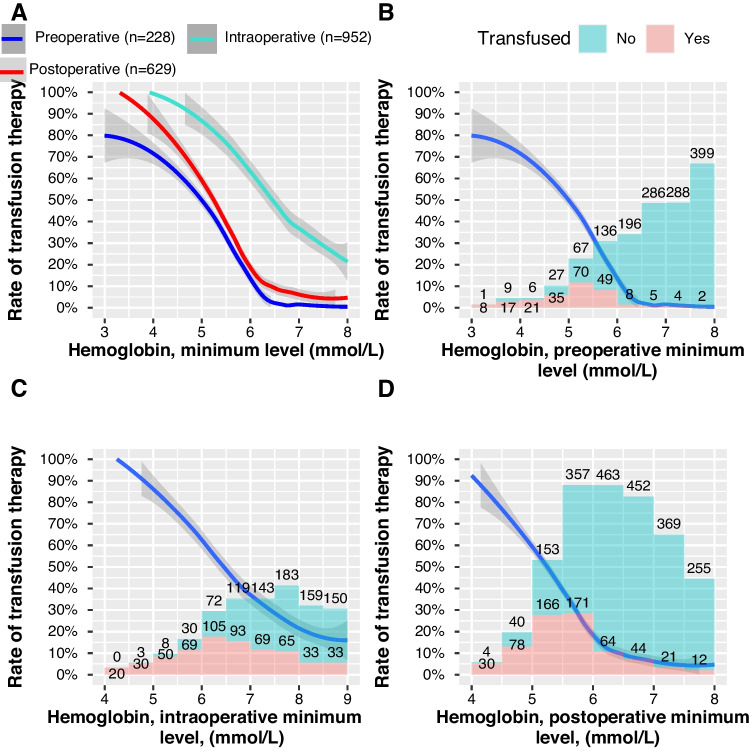
Transfusion rates in the stages of open abdominal surgery. **A** The rates of blood transfusion in the pre- (blue), intra- (turqoise), and post-operative phase of surgery (red). 95% confidence intervals depicted as the gray area around the curves. **B**, **C**, **D** The rates of transfusion in the pre-, peri-, and post-operative phase of surgery respectively, along with the number of patients in a histogram

Figure 4 A displays the rate of patients receiving a blood transfusion with a given minimum hemoglobin measurement. Figure 4 B shows that the 228 patients (7%) that received preoperative blood transfusions have received them at lower hemoglobin levels following a more restrictive strategy than for the peri- or post-operative phase (Fig. 4C and D respectively). The median minimum hemoglobin level preoperatively of those receiving blood was 5.2 mmol/L (interquartile range [IQR], 4.6–5.5). A total of 952 patients (30%) received blood transfusions intraoperatively. The median minimum hemoglobin level intraoperatively for receiving blood was 6.5 mmol/L (IQR, 5.7–7.5). The median minimum hemoglobin level postoperatively was 5.5 mmol/L (IQR, 5.1–5.9) in the 629 patients (20%) who received blood transfusion postoperatively. A total of 145 (23%) of the patients who received blood postoperatively had a postoperative hemoglobin level of 6 mmol/L or above. A total of 104, 17% of postoperatively transfused patients, had not had a single hemoglobin measurement below 6.0 mmol/L either pre- or intra-operatively.

### Impact of blood transfusions

There was a lower rate of mortality in the patients who did not receive transfusions in the intra- or postoperative phase with hemoglobin levels above 7 mmol/L and 6 mmol/L respectively.

**Fig. 5:**
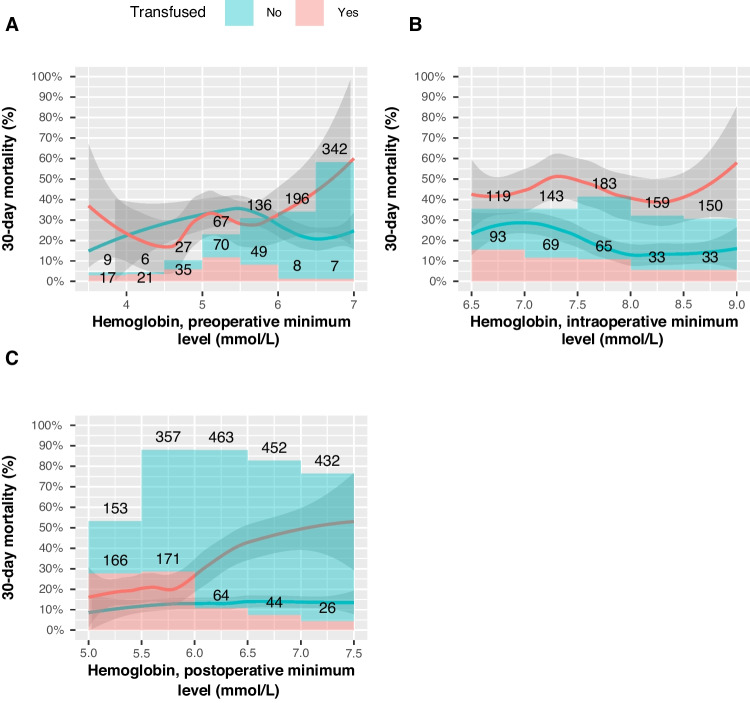
Mortality rates between transfused and non-transfused patients with respect to minimum pre- (**A**), intra- (**B**), and post-operative (**C**) hemoglobin level. Number of patients are shown as histograms. 95% confidence interval depicted as the gray area around the curve

Postoperatively, in the non-transfused patients, there is an approximately constant flat rate of mortality, despite having a low hemoglobin level postoperatively. The mortality of the patients who received transfusions increased and was significantly higher compared to the patients with the same hemoglobin level who were not transfused (Fig. 5C).

### Logistic regression prediction

**Fig. 6:**
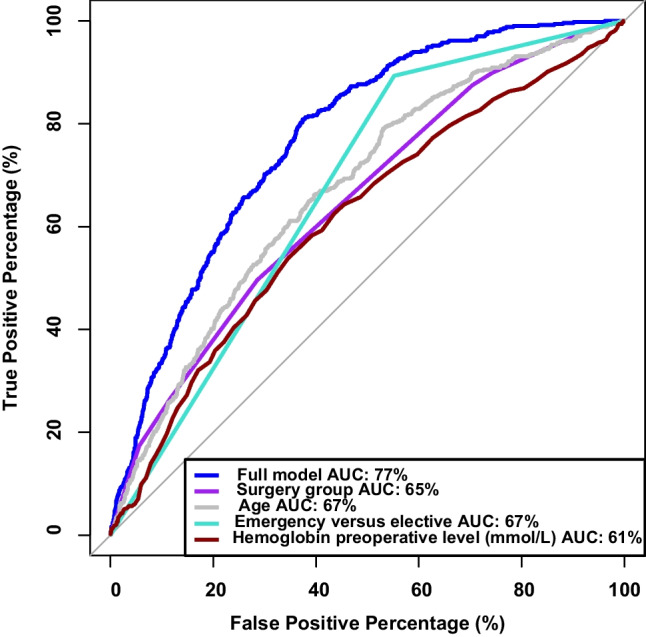
Receiver operating characteristic curve showing the prediction of 30-day mortality by individual variables and the final predictive model including all the individual variables on survival as determined by logistic regression. Area under the curve (AUC) of 50% indicates no difference from chance, and AUC of 100% indicates 100% sensitivity and 100% specificity. Surgical priority was either elective or emergency

The logistic regression model was constructed using four variables: operation group (categorized into 7 subtypes), age (continuous), the distinction between acute and elective surgery (binary), and preoperative hemoglobin levels (continuous). The performance of the model in predicting the 30-day survival status was 77% using the area under the receiver operating curve (AUC) (Fig. 6). Notably, preoperative hemoglobin was identified as a significant predictor (*P* < 0.05) after adjusting for all other variables. The odds ratios for the factors in the model along with confidence intervals and *P*-values can be seen from Table 2.

**Table 2:** Factors included in the logistic regression model

Variable	Odds ratio	95% confidence interval	*P*-value
Age (per year)	1.05	1.04–1.06	< 0.001
Hemoglobin, preoperative minimum level (per mmol/L)	0.87	0.81–0.94	< 0.001
Elective versus emergency surgery	0.18	0.13–0.25	< 0.001
Surgical category			
Gastroduodenal	3.64	1.83–7.27	< 0.001
Biliary	0.51	0.21–1.25	0.140
Small bowel	2.00	0.99–4.07	0.055
Colon	1.89	0.99–3.62	0.053
Rectum	1.97	0.95–4.09	0.067
Miscellaneous*	2.45	1.27–4.74	0.007

Reference for surgery type is appendicectomy (odds ratio 1.00)

*Herniotomy, adhesiolysis, splenectomy, or diagnostic procedures

## Discussion

This study demonstrated that a low preoperative hemoglobin level was significantly associated with increased mortality of patients undergoing open abdominal surgery. This association was linear, as mortality correlated negatively with the preoperative hemoglobin level. The logistical regression model performed demonstrated that the preoperative hemoglobin level independently added accuracy in determining those at risk of dying in relation to open abdominal surgery, on top of other risk factors like age and type of surgery.

These results are consistent with findings in other studies [5, 15, 16]. Duron et al. studied risk factors for elderly patients undergoing major abdominal surgery and found anemic patients to have an increased odds ratio of death of 1.80. They also find that within categories of people with the same disease, such as colon cancer, those with anemia had worse outcomes. A higher mortality was also found in those with only moderate anemia [15]. Mussalam et al., in a study on effects of preoperative anemia in non-cardiac surgery, found that 30% of patients had preoperative anemia, with an elevated odds ratio of 1.42 of dying. This was consistent across mild and moderate-to-severe anemia. Importantly, the study also found that the influence of anemia was additional to other risk factors [16]. Hemoglobin is a readily available biomarker for patients admitted for surgery.

Conflicting reports exist as to the benefits of restrictive transfusion practices. A meta-analysis from Roman et al. including a wide variety of surgery types reported that patient blood management interventions reduce bleeding and transfusion requirement in patients undergoing major surgery, but postoperative morbidity and mortality were not significantly reduced [17]. Acheson et al. reported that allogeneic blood transfusion (ABT) to patients operated on for colorectal cancer was associated with adverse clinical outcomes, including increased mortality, warranting measures aimed at limiting the use of ABT [11].

Our study showed a significant proportion of blood transfusions were given to patients with hemoglobin levels above what guidelines recommend. Figure 4 A shows that patients are most likely to be transfused intraoperatively, followed by the postoperative phase, whereas the preoperative phase had one fourth of the transfusions seen in the intraoperative phase.

The patients who received blood transfusions at close to normal hemoglobin levels contrary to a restrictive strategy showed increased mortality when compared to those not receiving blood transfusion, supporting reports such as Acheson et al. [11] that blood transfusion was associated with increased morality. This difference, or gap, in mortality between the transfused and non-transfused patients increased the closer to normal the patients’ hemoglobin levels were. Transfused patients had higher mortality, which is unsurprising, as transfusion may be confounded by intraoperative blood loss. The finding that the difference in mortality increased, the closer to normal, patient hemoglobin values were, could indicate an excess risk of mortality when these patients are subject to blood transfusion compared to the patients at low hemoglobin levels, one possible explanation being the benefits of blood transfusion are diminished as hemoglobin approaches normal levels, and the downsides, such as fluid overload, remain. Thus, blood transfusions, despite restrictive guidelines stating the hemoglobin level does not justify it, carry at best no benefit, and indeed from our data the patients seem to do worse than expected.

The strengths of this study included the large number of patients and data completeness, including hemoglobin level assessments and blood transfusion therapy across each patient’s perioperative course. Also, due to the unique civil registration number, no patients were lost to follow-up resulting in accurate reporting of 30-day mortality. A limitation was the lack of data on patient comorbidity that precluded a statistical adjustment for this important confounder. Details on comorbidity, ASA stage, and history leading up to the surgery has an effect on the mortality and thus the results should be interpreted with caution. The cohort study was retrospective and inherently, this makes causality unprovable. Moreover, as the study took place in a single center, the findings might not be generalizable to other surgical settings.

In conclusion, the preoperative hemoglobin level is significantly different preoperatively among those who survive compared to those who die within 30 days undergoing open abdominal surgery. There is a significant and linear association of lower values being associated with a higher mortality. A considerable fraction of patients received blood transfusions at hemoglobin levels above guideline recommendations. These patients have a higher mortality than patients who avoided transfusion.

## Data Availability

The data that support the findings of this study are available from the corresponding author, H. Buhl, upon reasonable request.
